# Integrative Features of the Yeast Phosphoproteome and Protein–Protein Interaction Map

**DOI:** 10.1371/journal.pcbi.1001064

**Published:** 2011-01-27

**Authors:** Nozomu Yachie, Rintaro Saito, Naoyuki Sugiyama, Masaru Tomita, Yasushi Ishihama

**Affiliations:** 1 Institute for Advanced Biosciences, Keio University, Tsuruoka, Japan; 2 Systems Biology Program, Graduate School of Media and Governance, Keio University, Fujisawa, Japan; 3 Department of Biological Chemistry and Molecular Pharmacology, Harvard Medical School, Boston, Massachusetts, United States of America; 4 Systems Biology Program, Faculty of Environment and Information Studies, Keio University, Fujisawa, Japan; 5 PRESTO, Japan Science and Technology Agency, Tokyo, Japan; Bar Ilan University, Israel

## Abstract

Following recent advances in high-throughput mass spectrometry (MS)–based proteomics, the numbers of identified phosphoproteins and their phosphosites have greatly increased in a wide variety of organisms. Although a critical role of phosphorylation is control of protein signaling, our understanding of the phosphoproteome remains limited. Here, we report unexpected, large-scale connections revealed between the phosphoproteome and protein interactome by integrative data-mining of yeast multi-omics data. First, new phosphoproteome data on yeast cells were obtained by MS-based proteomics and unified with publicly available yeast phosphoproteome data. This revealed that nearly 60% of ∼6,000 yeast genes encode phosphoproteins. We mapped these unified phosphoproteome data on a yeast protein–protein interaction (PPI) network with other yeast multi-omics datasets containing information about proteome abundance, proteome disorders, literature-derived signaling reactomes, and *in vitro* substratomes of kinases. In the phospho-PPI, phosphoproteins had more interacting partners than nonphosphoproteins, implying that a large fraction of intracellular protein interaction patterns (including those of protein complex formation) is affected by reversible and alternative phosphorylation reactions. Although highly abundant or unstructured proteins have a high chance of both interacting with other proteins and being phosphorylated within cells, the difference between the number counts of interacting partners of phosphoproteins and nonphosphoproteins was significant independently of protein abundance and disorder level. Moreover, analysis of the phospho-PPI and yeast signaling reactome data suggested that co-phosphorylation of interacting proteins by single kinases is common within cells. These multi-omics analyses illuminate how wide-ranging intracellular phosphorylation events and the diversity of physical protein interactions are largely affected by each other.

## Author Summary

To date, high-throughput proteome technologies have revealed that hundreds to thousands of proteins in each of many organisms are phosphorylated under the appropriate environmental conditions. A critical role of phosphorylation is control of protein signaling. However, only a fraction of the identified phosphoproteins participate in currently known protein signaling pathways, and the biological relevance of the remainder is unclear. This has raised the question of whether phosphorylation has other major roles. In this study, we identified new phosphoproteins in budding yeast by mass spectrometry and unified these new data with publicly available phosphoprotein data. We then performed an integrative data-mining of large-scale yeast phosphoproteins and protein–protein interactions (complex formation) by an exhaustive analysis that incorporated yeast protein information from several other sources. The phosphoproteome data integration surprisingly showed that nearly 60% of yeast genes encode phosphoproteins, and the subsequent data-mining analysis derived two models interpreting the mutual intracellular effects of large-scale protein phosphorylation and binding interaction. Biological interpretations of both large-scale intracellular phosphorylation and the topology of protein interaction networks are highly relevant to modern biology. This study sheds light on how *in vivo* protein pathways are supported by a combination of protein modification and molecular dynamics.

## Introduction

Protein phosphorylation is a reversible, ubiquitous, and fundamentally post-translational modification (PTM) that regulates a variety of biological processes; one of its critical roles is the control of protein signaling [1]–[3]. Recent advances in mass-spectrometry (MS)–based technologies and phosphopeptide enrichment methods have enabled the use of high-throughput *in vivo* phosphosite mapping [4]–[7] to identify thousands of phosphoproteins. To date, around 10,000 phosphosites of serine, threonine, or tyrosine residues have been identified in each of many organisms, including human [8]–[12], mouse [13] and yeast [14]–[16]. Many public databases, such as PHOSIDA [17], Phospho.ELM [18], and UniProt [19], have been developed or expanded to catalog such phosphoproteome data. Accordingly, the numbers of phosphoproteins that have been identified in various organisms now greatly exceed the numbers known to have roles in protein signaling. This has raised the question of whether this intracellular phosphorylation, which occurs on such a large scale, has other major roles.

In modern biology, the use of high-throughput screening methods has enabled rapid progress in the disclosure of protein–protein interaction (PPI) networks in many organisms [20]–[27]. Topological features common to PPI networks (e.g., scale-free and small-world properties) are of prime importance in interpreting intracellular protein behavior and the evolutionary aspects of PPIs [28]–[31]. PTM changes the physical characteristics of proteins. It is therefore probable that reversible PTM has large effects on the dynamic states of intracellular protein-binding patterns and complex formation, and that it controls not only signal transduction but also many other cellular pathways. However, the impact of PTM on the whole picture of the PPI network has not yet been described.

**Figure 1:**
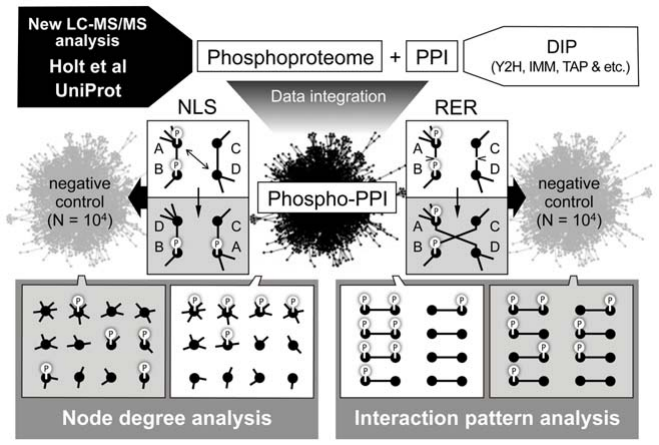
Overview of integrative analysis of yeast multi-omics data. New phosphoproteins were identified by LC-MS/MS analysis and unified with the publicly available phosphoproteome datasets of Holt et al. [16] and UniProt [19] (Step 1). A protein–protein interaction (PPI) map was obtained from DIP (Database of Interacting Proteins) [33] (Step 2). Y2H, yeast two-hybrid; IMM, co-immunoprecipitation; TAP, tandem affinity purification. The “phospho-PPI” map was generated by superimposing the phosphoproteome data onto the PPI map (Step 3). Negative controls for the phospho-PPI map were generated by “node label shuffling (NLS)” and “random edge rewiring (RER)” (Step 4). Comparative analyses of the real phospho-PPI and its negative controls were performed with other yeast multi-omics data (Step 5).

Here, we describe the intracellular global relationships between protein phosphorylation and physical PPI, as derived from the results of integrative and systematic data-mining of *Saccharomyces cerevisiae* multi-omics data (Fig. 1). New phosphoproteome data on *S. cerevisiae* were initially obtained by MS–based analysis and unified with data on previously identified phosphoproteomes. We superimposed the unified phosphoproteome data onto a *S. cerevisiae* PPI network with other multi-omics data on *S. cerevisiae.* From the results, we infer that the tremendous numbers of phosphorylations within a cell have a large impact on PPI diversity, and that intracellular phosphorylation patterns are affected partly by simultaneous phosphorylation of physically bound proteins that is triggered by the action of single kinases.

## Results/Discussion

### New yeast phosphoproteome data

**Figure 2:**
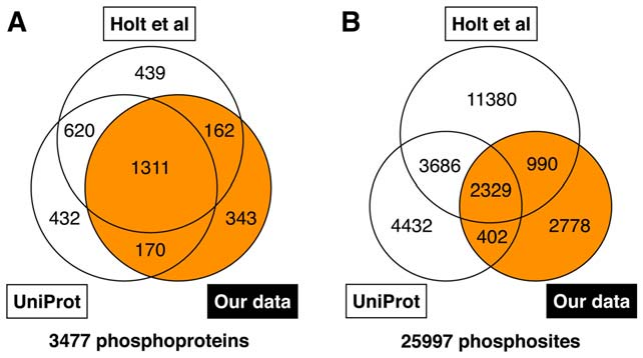
Number counts of phosphoproteins (A) and their phosphosites (B) newly identified in this study and of those obtained from the data of Holt et al. [**16**] and UniProt [**19**].

On the basis of liquid chromatography (LC)-MS analysis, we initially identified 1,993 *S. cerevisiae* phosphoproteins containing 6,510 phosphosites. Information on the identified phosphopeptides has been stored in PepBase (http://pepbase.iab.keio.ac.jp). We unified these new phosphoproteome data with the publicly available phosphoproteome datasets of Holt et al. [16] and UniProt [19] and obtained a total of 3,477 phosphoproteins containing 25,997 phosphosites (Fig. 2; Supplementary Table S1). The pS/pT/pY ratios of this study, the study of Holt et al., and UniProt were 72%/23%/5%, 72%/23%/5%, and 80%/18%/2%, respectively. Among the unified phosphoproteome data, 343 phosphoproteins and 2,778 phosphosites were not found in the data of Holt et al. or UniProt. Comparison with *S. cerevisiae* genomic information [32] revealed that 58.5% of the 5,815 known and predicted genes were phosphoprotein-encoding genes (Supplementary Table S2). Although the use of current high-throughput technologies cannot disclose the entire phosphoproteome picture of a cell, these results imply that most intracellular proteins can be phosphorylated under the appropriate environmental conditions.

### Generation of phospho-PPI network

The unified phosphoproteome data were superimposed onto the PPI network to generate a “phospho-PPI” network. PPI data were obtained via DIP (Database of Interacting Proteins) [33] and grouped into four categories according to the experimental method used for the PPI assay: all kinds of experimental methods (“ALL”), yeast two-hybrid (“Y2H”), co-immunoprecipitation (“IMM”), and tandem affinity purification (“TAP”). Among all the protein nodes involved in every category of the phospho-PPI network, the proportion of phosphoproteins was also nearly 60% (Supplementary Fig. S1). For example, the phospho-PPI network of the “ALL” category was composed of 4,945 proteins, including 2,934 phosphoproteins (59.3%) and 17,215 physical interactions.

### Phosphoproteins have more PPI partners than do nonphosphoproteins

**Figure 3:**
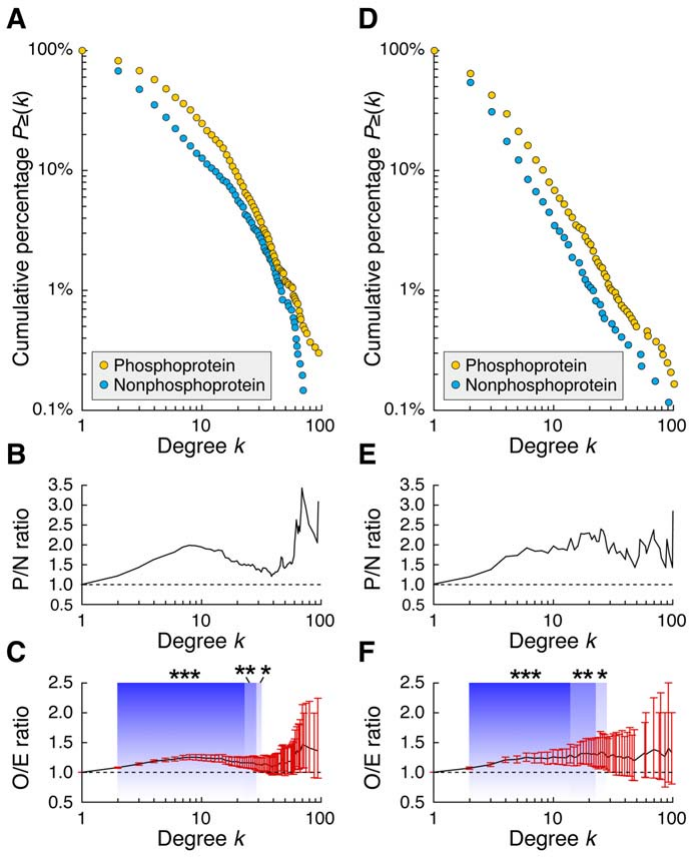
Node degree distributions of phosphoproteins and nonphosphoproteins in the phospho-PPI data sets of “ALL” (A–C) and “Y2H” (D–F). (**A,D**) Cumulative probability distribution of node degrees. For each group of phosphoproteins and nonphosphoproteins, circles represent proportions of proteins with more than the *k* interacting partners indicated on the horizontal axis [*P*≥(*k*)]. (**B,E**) P to N (P/N) ratio, where P and N are *P*≥(*k*) of phosphoproteins and nonphosphoproteins, respectively. (**C,F**) O to E (O/E) ratio, where O is *P*≥(*k*) of phosphoprotein and E is that expected from negative controls generated by node label shuffling (N = 10,000). For each node degree level, line graph and bar represent mean and two-sided 95% confidence intervals, respectively. Background colors and asterisks denote statistical significance over neutral O/E value of 1.0 (*P<0.05, **P<0.01, ***P<0.001).

To explore specific characteristics of the phospho-PPI network, the number counts of interacting partners of phosphoproteins and nonphosphoproteins were analyzed (note that throughout this study, the word “nonphosphoprotein” means a protein with no phosphosite identified to date). We found that, in general, phosphoproteins had more interacting partners than nonphosphoproteins. In each phospho-PPI network of the “ALL” and “Y2H” categories with enough protein nodes for the subsequent statistical analysis, the cumulative percentage distributions of node degrees (or the number count of interacting partners) of phosphoproteins and nonphosphoproteins were markedly different (Fig. 3A and D). For example, in the dataset of “ALL”, 47.6% of nonphosphoproteins had three or more interacting partners, but this was true for 67.9% of phosphoproteins. Moreover, in both datasets, about twice as many phosphoproteins as nonphosphoproteins had 10 interacting partners (Fig. 3B and E). To analyze the statistical significance of this difference in the context of phosphorylation, we prepared randomly generated phospho-PPI networks by “node label shuffling” (NLS), in which the node positions of phosphoproteins and nonphosphoproteins were randomly moved within the phospho-PPI networks (for details, see Materials and Methods). This demonstrated that the node degree of phosphoproteins was significantly higher than expected from a random distribution (Fig. 3C and F).

**Figure 4:**
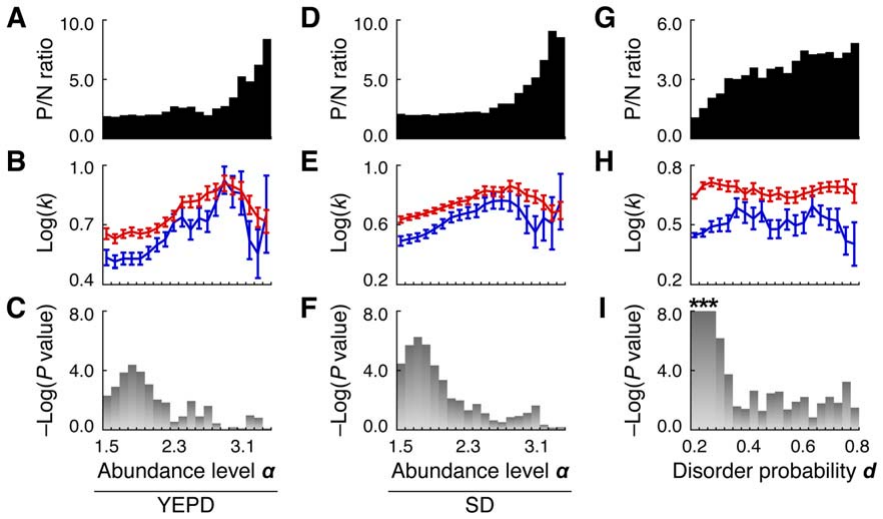
Difference between node degree levels of phosphoproteins and nonphosphoproteins at each level of protein abundance (A–F) and protein disorder (G–I). (**A–C**) YEPD, protein abundance dataset for cells grown in rich medium; (**D–F**) SD, protein abundance dataset for cells grown in synthetic complete medium. Protein abundance provided in the original dataset [37] was log-transformed (base 10) as abundance level α. The type of phospho-PPI network is “ALL” (for each analysis, protein nodes for which abundance or disorder levels and their corresponding edges were not provided were eliminated from the phospho-PPI network). Each bin corresponds to the protein abundance level between α (indicated on the horizontal axis) and α+0.5 (**A–F**) or the protein disorder probability between *d* (indicated on the horizontal axis) and *d*+0.2 (**G–I**). For protein nodes corresponding to each bin, the P to N (P/N) ratio of protein number count (where P and N are number counts of phosphoproteins and nonphosphoproteins) (**A,D,G**), average node degree levels [Log(*k*) (base 10)] of phosphoproteins (red line) and nonphosphoproteins (blue line) (**B,E,H**), and statistical significance [−Log(*P* value) (base 10)] of differences between Log(*k*) of phosphoproteins and nonphosphoproteins (**C,F,I**) are represented. Error bars denote s.e.m. Asterisks denote −Log(*P* value)>8.0 (i.e. *P*<10^−8^).

Node degree in PPI networks has an exponential relationship with protein expression level [34]–[36], perhaps because cellular proteins with more copies have a greater possibility of interacting with others by chance [36]. Therefore, if the phosphoproteome data are biased by protein abundance and highly abundant proteins tend to be identified as phosphoproteins, there is a strong possibility that the relationship between phosphorylation and node degree is spurious, with no direct causal connection. In fact, proteome abundance data obtained through a single-cell proteomic analysis combining high-throughput flow cytometry and a library of GFP-tagged yeast strains [37] showed that the number of phosphoproteins in the “ALL” phospho-PPI was skewed, especially among highly abundant proteins (Fig. 4A and D). However, we demonstrated that in the “ALL” phospho-PPI network there were still significant differences in the node degree levels of phosphoproteins and nonphosphoproteins of similar abundance, and that the differences could be explained independently of protein copy number (Fig. 4B, C, E and F). Similar results were derived from the phospho-PPI network generated only from the “Y2H” category (Supplementary Fig. S2). We further compared the abilities to predict phosphoproteins by using node degree and protein abundance levels above given thresholds. The predictive power of node degree was markedly higher than that of protein abundance, except in the case of proteins that were extremely abundant (Supplementary Fig. S3). If this higher predictive ability were attributable to a spurious relationship associated with the actual intracellular proteome abundance, then the node degree of a protein given by PPI assays would appear to provide a better approximation of the intracellular protein copy number than would single-cell proteomic analysis, which is unlikely.

Protein disorder is also a typical feature of “hub” proteins in PPI networks [38]–[40]. Parts of unstructured proteins lack fixed structure, and such disordered regions may have the ability to bind multiple proteins and to diversify PPI networks [38]–[40]. Additionally, at the proteome level, phosphorylation occurs at high rates in the disordered regions of proteins [16], [17], [41]–[44]. Therefore, it is highly likely that protein disorder affects the node degree difference between phosphoproteins and nonphosphoproteins. For every *S. cerevisiae* protein registered in UniProt, we calculated the probability of harboring intrinsic disordered regions (see Materials and Methods). In the “ALL” phospho-PPI network, the ratio of phosphoproteins to nonphosphoproteins increased smoothly with increasing disorder probability level (Fig. 4G). However, in the same network, the node degree levels of phosphoproteins and nonphosphoproteins of the same disorder probability level were significantly different (Fig. 4H and I). Even between phosphoproteins that had a low disorder probability of <0.1 and nonphosphoproteins that had an extremely high disorder probability of >0.9, the node degree level of the phosphoproteins was significantly higher than that of the nonphosphoproteins (*P* = 0.0043). Similar results were observed in the “Y2H” dataset (Supplementary Fig. S2). These results imply that the higher node degree of phosphoproteins than of nonphosphoproteins is at least partly independent of the PPI network diversity produced by unstructured proteins.

Other factors that could influence the relationship between protein phosphorylation and interaction are protein size and protein groups with identical cellular function. Larger proteins may have a greater chance of being phosphorylated and may provide more binding domains for interactions with other proteins. However, similar to the results for protein abundance and disorder, statistical significance of the higher node degree of phosphoproteins was observed independently of protein length (Supplementary Fig. S4). (Phosphorylation probability was highly correlated with protein length; Supplementary Fig. S4.) In the event that both protein phosphorylation and interaction events occurring in a fraction of proteins confer a particular, identical cellular function, then the global difference in node degree levels of phosphoproteins and nonphosphoproteins would appear to be caused only by differences in function. However, we found that, for most functional annotations of *S. cerevisiae* in GO Slim (a higher level view of Gene Ontology), there was a higher node degree level for phosphoproteins than for nonphosphoproteins (Supplementary Fig. S5).

The average node degree of phosphoproteins is higher than that of nonphosphoproteins [45], but it was unclear 1) whether this characteristic was observable only in hub proteins or whether it existed broadly at the proteome level; and 2) whether this was a spurious correlation that had emerged because of the presence of some third factor hidden in the complex and intertwining proteomes. Our results show that, in many cases, this characteristic is present not only in hub proteins but also in proteins that have few interacting partners. They also imply that these protein interactions or binding patterns are not the result of influence by a third factor but are caused by phosphorylation-dependent cellular activities.

### Diversification of PPIs by phosphorylation

The additive effect of kinase–substrate and phosphatase–substrate reactions is one possible model for interpreting this phenomenon in the phospho-PPI network. If PPIs include many transient signaling reactions between kinases, phosphatases, and their substrates (most of which are phosphorylated under certain conditions), then the signaling proteins may have interactions additional to the cohesive protein binding interactions in the PPI data. Indeed, some enzyme–protein substrate interactions are surprisingly stable and can be captured in protein interaction assays [46]. However, of the 795 yeast phosphorylation and dephosphorylation reactions for which information has previously been published [47], only 3.9%, 1.6%, 2.4%, and 0.8% overlapped with those in our “ALL,” “Y2H,” “IMM,” and “TAP” PPI datasets, respectively (Supplementary Fig. S6). [Note, however, that these values were significantly higher than those expected from negative controls of the corresponding PPI networks generated by “random edge rewiring” (RER), and similar, significant overlaps between physical PPI and signaling network were obtained by another group [48]; for details of RER, see Materials and Methods.] On the other hand, the node degree levels of at least 600 proteins (>20% of phosphoproteomes in the “ALL” phospho-PPI network) might have been related to, and affected by, phosphorylation, as evidenced by the cumulative percentage of phosphoproteins, which was more than 20% higher than that of nonphosphoproteins (Fig. 3A). In addition to this, many unidentified phosphoproteins are certain to be present in the nonphosphoprotein dataset. Therefore, it is difficult to interpret such a large difference in the node degree of phosphoproteins and nonphosphoproteins only in terms of the additive effect of signaling reactions, which had such a small overlap with the PPI data. Furthermore, among the GO Slim ontology groups within the “signal transduction” and “cell cycle” categories, which especially include many signaling proteins, there were no great distinctions between the node degree levels of phosphoproteins and nonphosphoproteins (although the node degree levels for “cytokinesis” and “response to stress,” like those for most of the other ontology groups, showed marked differences between phosphoproteins and nonphosphoproteins) (Supplementary Fig. S5).

**Figure 5:**
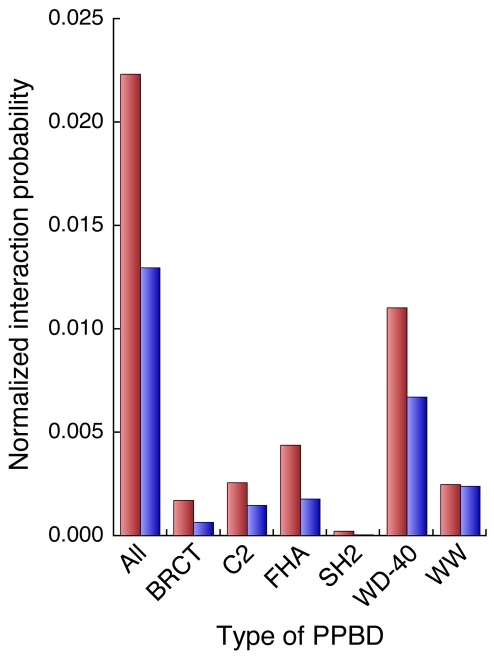
Probabilities that phosphoproteins and nonphosphoproteins will interact with proteins that have phosphoprotein binding domains (PPBDs). The average normalized interaction probabilities of phosphoproteins (red bars) and nonphosphoproteins (blue bars) in the “ALL” phospho-PPI network with each type of PPBD or with all PPBDs (indicated on the horizontal axis) are shown.

In the phospho-PPI network, phosphoproteins had a greater tendency than nonphosphoproteins to interact with proteins harboring phosphoprotein binding domains (PPBDs). Out of 10 known PPBDs—14-3-3, BRCT, C2, FHA, MH2, PBD, PTB, SH2, WD-40, and WW [49]—six (BRCT, C2, FHA, SH2, WD-40, and WW) were present in the member proteins of the “ALL” phospho-PPI network, and the average probabilities that phosphoproteins would interact with proteins that had all PPBDs or each type of PPBD were higher than those for nonphosphoproteins (Fig. 5). (The gap between node degree levels of phosphoproteins and nonphosphoproteins was normalized; see Materials and Methods.) Considering all of these results and perspectives, a reasonable and generalized model that can be used to interpret the higher node degree of phosphoproteins is that reversible and alternative phosphorylation reactions alter the physical characteristics of proteins under various environmental conditions; the interacting or binding partners of phosphoproteins are thereby more diversified than those of nonphosphorylated proteins. Consistent with this interpretation, phosphoproteins harboring at least two phosphosites had more interacting partners than those with a single phosphosite in the phospho-PPI network (Supplementary Fig. S7), even though phosphoproteins follow a power-law distribution with regard to phosphosite number counts and only a small fraction of phosphoproteins have multiple phosphosites [50]. Protein phosphorylation reactions therefore seem to make a large contribution to intracellular PPI diversity.

### Both interacting proteins tend to be phosphorylated

**Figure 6:**
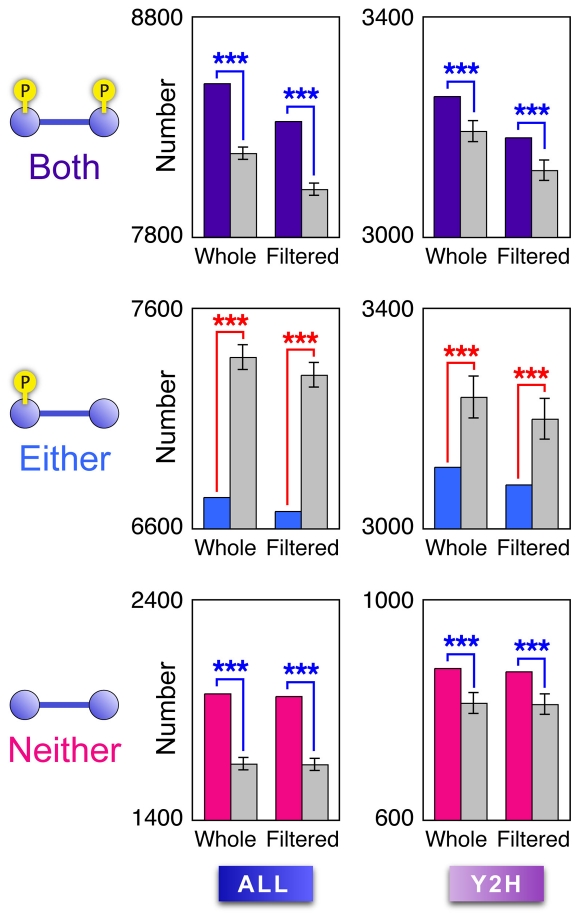
Number counts of interacting protein pairs of each phosphorylation pattern shown in the phospho-PPI network. Respective rows of panels correspond to the three phosphorylation patterns of two interacting proteins: “Both” (**A,B**) “Either” (**C,D**) and “Neither” (**E,F**) respective columns correspond to PPI categories of “ALL” (**A,C,E**) and “Y2H” (**B,D,F**). In each panel, data are shown for two types of phospho-PPI networks: “whole” (i.e. unfiltered) and “filtered” (see text). Colored bars (purple, blue, and pink) represent number counts of protein interactions in real data sets; gray bars show mean values of those estimated by negative controls generated by random edge rewiring (N = 10,000). Error bars represent s.d. Blue/red asterisks denote significance of values higher/lower than those of negative controls (***P<0.001).

We further analyzed the phosphorylation patterns of protein pairs forming pair-wise interactions in the phospho-PPI network, and we found that both interacting proteins in each pair tended to be phosphorylated. For every category of phospho-PPI network, three types of pair-wise interactions were counted, whereby “Both,” “Either,” or “Neither” of two interacting proteins were phosphorylated. The “Both” and “Neither” types of protein interactions were significantly more common in the real phospho-PPI network than was expected from negative controls produced by RER, whereas the “Either” types of protein interactions were significantly less common than expected (Fig. 6; Supplementary Fig. S8). Notably, this outcome was independent of whether the node degrees of the phosphoproteins were higher or lower than those of the nonphosphoproteins, because RER does not change the node degree of each protein in a given network [51].

PPI data contain homodimer and heterodimer information that can be captured by experimental assays such as two-hybrid assays [52]. Therefore, to check the possibility that the tendency of interacting proteins to have similar phosphorylation patterns was caused by protein interactions between structurally and sequentially homologous proteins with similar phosphosites, we conducted the same analysis as above but using “filtered” phospho-PPI networks, in which interactions between two homologous proteins were eliminated by E-value cut-offs of 1e–10 in the BLASTP program, but no marked change was observed (Fig. 6; Supplementary Fig. S8).

### Co-phosphorylation of interacting proteins by single kinase

**Figure 7:**
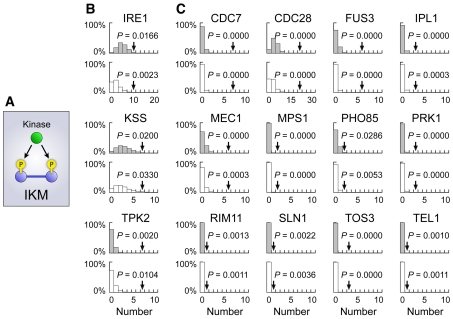
Kinases inferred to yield co-phosphorylation of proteins in the same protein complex. (**A**) Conceptual diagram of an interacting kinate module (IKM) motif. (**B,C**) Kinases revealed to have significantly higher IKM formability than negative controls by data integration of the PPI network with *in vitro* kinase–substrate relationships (**B**) and with a literature-based collection of signaling pathways (**C**). For each kinase, arrows denote number counts of IKMs formed by that kinase and the “whole” (i.e. unfiltered) PPI network, with *P* values estimated from negative controls of the PPI network generated by RER and NLS (N = 10,000). Expected probability density distributions of number counts of IKMs observed in negative controls generated by node label shuffling and random edge rewiring are shown by gray and white bars, respectively.

Proteins involved in signal transduction pathways tend to be phosphorylated, and this is reflected in the PPI data, although the overlaps between such signaling reactions and PPIs are limited (see above and Supplementary Fig. S6). Another possible interpretation for the multitude of physical interactions between phosphoproteins is that physically binding proteins that are members of the same protein complex tend to be phosphorylated simultaneously by a single enzyme. To search for the protein kinases potentially responsible for the co-phosphorylation of proteins forming the same complex, we analyzed a dataset of kinase–substrate relationships with PPI data of the “ALL” category. In the following analysis, we used 85 and 65 kinases, respectively, from the experimental results of an *in vitro* kinase–substrate assay [53] and a literature-derived collection of yeast signaling reactions [47], each having multiple substrates (Supplementary Table S3). For each kinase, its multiple substrates were superimposed on the PPI network and the number of “interacting kinate modules” (IKMs, triangle motifs composed of a kinase and its two physically interacting substrates) (Fig. 7A) [53] was counted and compared with those estimated in negative controls of the PPI network produced by NLS and RER. This analysis revealed that three kinases from the *in vitro* assay and 12 from the literature-based collection had significantly higher IKM formability than those expected from both NLS and RER (*P*<0.05) (Fig. 7B and C; Supplementary Table S3). Similar results were obtained by using the “filtered” phospho-PPI network (Supplementary Fig. S9; Supplementary Table S3).

Accordingly, we suggest that, when a protein complex and kinase are in close proximity within the intracellular environment, there is a high chance of simultaneous phosphorylation of member proteins participating in the complex. This is consistent with the subcellular co-localization of signaling networks recently revealed through the systematic prediction of signaling networks by using phosphoproteome data with an integrated protein network information derived from curated pathway databases, co-occurring terms in abstracts, physical protein interaction assays, mRNA expression profiles, and the genomic context [48], and by data analysis of time-course phosphoproteome data [54]. IKMs may enhance the subcellular co-localization of signaling reactions, and/or vice versa. The literature-derived signaling collection is presumably more enriched with well-investigated reactions and thus may more accurately reflect *in vivo* signaling. This may explain why the collection harbored more kinases with high IKM formabilities (12 out of 65) than the *in vitro* kinase–substrate relationship data (three out of 85).

### Bi-directional impacts of protein phosphorylation and binding

It is plausible that, in living cells, the diversity of protein interactomes (not only of protein signaling but also of protein complex formation) is essentially influenced by the large number of phosphorylation events; many reversible phosphorylations might control condition-specific protein binding interactions related to different subcellular processes and molecular machines. On the other hand, protein phosphorylation patterns also seem to depend largely on intracellular protein interaction diversity.

It is possible that many of the proteins defined as nonphosphoproteins in this study can actually be phosphorylated under appropriate cellular conditions. Even where this is true, however, the set we defined here as phosphoproteins should be enriched with proteins that are frequently phosphorylated under normal or many different cellular conditions, because the frequently phosphorylated proteins have a higher chance of being identified as phosphoproteins than do the rarely phosphorylated proteins. Accordingly, the features and models discussed in this study should reflect the overall characteristics of phosphoproteins and nonphosphoproteins among a number of different cellular conditions. This is supported by the finding that proteins that had two or more phosphosites physically interacted with more proteins than did those with only a single phosphosite (Supplementary Fig. S7). Although the quality of current yeast PPI data is also not perfect and the data may include false positives, the observed features with statistical significance should be consequences of the actual behaviors of intracellular proteins, because the effects of such false positives on the statistical tests are supposedly random.

The integrative data-mining of yeast multi-omics data has now shed light on the macroscopic and large-scale relationships between phosphoproteomes and protein interactomes. Future comprehensive analyses of the *in vivo* link between protein phosphorylation and physical interaction will yield more insights into the complex and intertwined molecular systems of living cells.

## Materials and Methods

### Phosphopeptide samples

*Saccharomyces cerevisiae* strain IFO 0233 cells grown continuously on glucose medium [55] were used. Pelleted cells were vacuum dried and frozen until further analysis. A Bioruptor UCW-310 (Cosmo Bio, Tokyo Japan) was used to disrupt the pellets in 0.1 M Tris-HCl (pH 8.0) containing 8 M urea, protein phosphatase inhibitor cocktails 1 and 2 (Sigma), and protease inhibitors (Sigma). The homogenate was centrifuged at 1,500*g* for 10 min and the supernatant was reduced with dithiothreitol, alkylated with iodoacetamide, and digested with Lys-C; this was followed by dilution and trypsin digestion as described [56]. Digested samples were desalted by using C-18 StageTips [57]. Phosphopeptide enrichment by hydroxy acid–modified metal oxide chromatography (HAMMOC) was performed as reported previously [11], [58]. Briefly, digested lysates (100 µg each) were loaded onto a self-packed titania-C8 StageTip in the presence of lactic acid. After the samples had been washed with 80% acetonitrile containing 0.1% TFA, phosphopeptides were eluted by a modified approach using 5% ammonium hydroxide, 5% piperidine, and 5% pyrrolidine in series [59].

### LC-MS/MS analysis

An LTQ-Orbitrap XL (Thermo Fisher Scientific, Bremen, Germany) coupled with a Dionex Ultimate 3000 (Germering, Germany) and an HTC-PAL autosampler (CTC Analytics AG, Zwingen, Switzerland) was used for nanoLC-MS/MS analyses. An analytical column needle with a “stone-arch” frit [60] was prepared with ReproSil C18 materials (3 µm, Dr. Maisch, Ammerbuch, Germany). The injection volume was 5 µL and the flow rate was 500 nL/min. The mobile phases consisted of (A) 0.5% acetic acid and (B) 0.5% acetic acid and 80% acetonitrile. A three-step linear gradient of 5% to 10% B in 5 min, 10% to 40% B in 60 min, 40% to 100% B in 5 min, and 100% B for 10 min was employed throughout this study. The MS scan range was *m*/*z* 300 to 1500, and the top 10 precursor ions were selected in MS scans by Orbitrap with R = 60,000 for subsequent MS/MS scans by ion trap in the automated gain control (AGC) mode; AGC values of 5.00e+05 and 1.00e+04 were set for full MS and MS/MS, respectively. The normalized collision energy was set at 35.0. A lock mass function was used for the LTQ-Orbitrap to obtain constant mass accuracy during gradient analysis.

### Phosphosite identification

Both Mass Navigator v1.2 (Mitsui Knowledge Industry, Tokyo, Japan) and Mascot Distiller v2.2.1.0 (Matrix Science, London, UK) were used to create peak lists based on the recorded fragmentation spectra. Peptides and proteins were identified by automated database searching using Mascot Server v2.2 (Matrix Science) against UniProt/SwissProt v56.0 with a precursor mass tolerance of 3 ppm, a fragment ion mass tolerance of 0.8 Da, and strict trypsin specificity, allowing for up to two missed cleavages. Carbamidomethylation of cysteine was set as a fixed modification, and oxidation of methionines and phosphorylation of serine, threonine, and tyrosine were allowed as variable modifications. Phosphopeptide identification and phosphorylated site determination were performed in accordance with a procedure reported previously [11]. The false discovery rate was estimated to be 1.07% using a randomized database. All annotated MS/MS spectra were stored in PepBase (http://pepbase.iab.keio.ac.jp).

### Public phosphoproteome datasets

*Saccharomyces cerevisiae* phosphoproteome data were obtained from Dataset S1 of Holt et al. [16]. Another collection of formerly identified phosphoproteins and their phosphosites was obtained from UniProt (release 15.14; http://www.uniprot.org/) [19]. All UniProtKB/Swiss-Prot protein entries identified to have at least one phosphosite in high-throughput phosphoproteomics studies were downloaded via the Protein Knowledgebase (UniProtKB) in XML format by querying the term scope: “PHOSPHORYLATION [LARGE SCALE ANALYSIS] AT”. Some phosphoproteins registered in UniProt had multiple synonyms of UniProt accession.

### Identifier standardization and data integration

For integrative analyses and comparisons of yeast multi-omics data, all identities of proteins and genes obtained from different data sources were standardized to UniProt accessions. If objects (e.g. gene names, ORF names, and/or locus names) in a data source did not have UniProt accessions, the objects were standardized to their corresponding UniProt accessions according to the cross-reference list prepared from UniProtKB/Swiss-Prot protein entries obtained from UniProt (release 15.14). In cases when an object corresponded to multiple synonyms of UniProt accessions, all accessions were used to identify its corresponding objects in other data sources.

### Phosphoproteome data unification

The phosphoproteome data newly identified in this study and the former phosphoproteome datasets obtained from Holt et al. and UniProt were unified according to their UniProt accessions. Positions of phosphosites and their amino acid residues in the unified phosphoproteome data were double-checked by using the proteome sequences obtained from UniProt (release 15.14).

### Gene annotations

From SGD (*Saccharomyces* Genome Database; http://yeastgenome.org) [32], annotations of 5,815 known and predicted genes were obtained. ORF names of genes were checked by using the unified phosphoproteome data to determine whether the encoded protein was identified as a phosphoprotein.

### PPI network

The *S. cerevisiae* PPI network was obtained as XML files (Scere20081014) from DIP (Database of Interacting Proteins; http://dip.doe-mbi.ucla.edu) [33]. We eliminated each interaction entry including three or more “interactors” (e.g., in which multiple prey proteins were detected for one bait protein in one experimental assay) and used only those including two “interactors.” Every node in the PPI network was labeled by its corresponding UniProt ID provided in the same XML file. For the PPI assay, PPI data were further grouped into four categories: all kinds of experimental methods (“ALL”), yeast two-hybrid (“Y2H”), co-immunoprecipitation (“IMM”), and tandem affinity purification (“TAP”). A “filtered” PPI network was also prepared for each category by eliminating interactions between two similar proteins by using the BLASTP program and an E-value cut-off of 1e–10.

### Phospho-PPI

Unified phosphoproteome data were mapped onto every category of PPI data prepared from DIP according to their UniProt accessions, and a phospho-PPI network was generated. Throughout this study, proteins that did not correspond to phosphoproteome data were termed “nonphosphoproteins.”

### Negative control generation

To prepare negative controls for PPI and phospho-PPI networks, two different processes (as diagrammed in Fig. 1) were appropriately adopted on a case-by-case basis. “Node label shuffling” (NLS) swaps the labels of two randomly selected nodes in a given network; it repeats this operation a sufficient number of times until all pair-wise interactions in the queried network have disappeared or until the number of iterations reaches 1,000 times the number of interactions. “Random edge rewiring” (RER) randomly selects two edges in a given network and randomly rewires them. During this process, each rewiring operation is retried if a pair of nodes redundantly wired by two edges occurs in the network; the iteration termination condition is the same as that of NLS.

### Proteome abundance data

Proteome abundance data for *S. cerevisiae* that were previously acquired through a single-cell proteomics analysis combining high-throughput flow cytometry and a library of GFP-tagged strains [37] were used to analyze the characteristics of protein expression in the phospho-PPI network. These data were composed of proteome abundance data measured for cells grown in rich (YEPD) and synthetic complete (SD) medium. For each cell growth condition, protein names were standardized to UniProt accessions, and protein abundance levels were log-transformed (base 10) and superimposed on each of the phospho-PPI networks of “ALL” and “Y2H.” In this case, protein nodes for which the abundance levels were not provided in the abundance data were deleted from the phospho-PPI network.

### Prediction of structured and unstructured proteins

The protein disorder level of every *S. cerevisiae* protein registered in UniProt (release 15.14) was predicted by the POODLE-W program, which uses the support vector machine–based learning of amino acid sequences of structurally confirmed disordered proteins [61]. For the analysis, we used the “disorder probability” (i.e. the probability that a given protein is unstructured) output by this program.

### Gene Ontology

*Saccharomyces cerevisiae* gene annotations belonging to “molecular function,” “biological process,” or “cellular component” of GO Slim, a higher level view of *S. cerevisiae* Gene Ontology (GO), were downloaded via the SGD ftp site.

### PPBD

Information on *S. cerevisiae* proteins, each of which has at least one of 10 known phosphoprotein binding domains (PPBDs), namely 14-3-3, BRCT, C2, FHA, MH2, PBD, PTB, SH2, WD-40, and WW [49], was obtained according to the protein domain annotations of UniProt (release 15.14), which were provided by other protein databases.

### Normalized probability of interaction with PPBDs

To evaluate the tendencies of phosphoproteins and nonphosphoproteins to interact with proteins that had PPBDs, the normalized probabilities of such interactions were defined. For each protein, the number of interacting protein partners that had PPBDs was divided by the number of all interacting partners.

### Collection of signaling reactions

To find possible IKMs, kinases previously reported to phosphorylate multiple substrates were obtained from data on *in vitro* substrates recognized by most yeast protein kinases that were measured with the use of proteome chip technology [Supplementary Data 2 of Ptacek et al. [53]], as well as from a literature-derived collection of documented yeast signaling reactions [Table S3 of Fiedler et al. [47]]. All gene names of substrates in the *in vitro* kinase–substrate relationship data and ORF names of substrates in the literature-derived collection were standardized into UniProt accessions and linked to proteins in the “whole” and “filtered” PPI networks of the “ALL” category.

### Statistics

The statistical significance of differences in a single real value from a group of repeatedly generated random values was estimated by calculating the proportion of random values equal to the real value or more (or less, in certain instances). The Wilcoxon–Mann-Whitney rank sum was used to assess statistical significance between groups.

## Supporting Information

Figure S1 Contents of the phospho-PPI network generated for each experimental method used in the PPI assay. Numbers in parentheses indicate those derived by the “filtered” network.
(1.73 MB TIF)

Figure S2 Differences between node degree levels of phosphoproteins and nonphosphoproteins of the “Y2H” phospho-PPI network at each level of protein abundance or protein disorder. See legend to Fig. 4 for details.
(0.67 MB TIF)

Figure S3 Comparison of abilities to predict phosphoproteins by node degree level and protein abundance level. (A) “ALL” PPI data and proteome abundance data measured for cells grown in YEPD medium. (B) “ALL” PPI and proteome abundance for cells grown in SD medium. (C) “Y2H” PPI and proteome abundance for cells grown in YEPD medium. (D) “Y2H” PPI and proteome abundance for cells grown in SD medium. For each predictor, the true-positive rate or “sensitivity” (defined here as the fraction of phosphoproteins correctly predicted to be phosphoproteins) and the false-positive rate or “1 – specificity” (defined here as the fraction of nonphosphoproteins incorrectly predicted to be phosphoproteins) are shown at a series of score thresholds.
(5.75 MB TIF)

Figure S4 Differences between node degree levels of phosphoproteins and nonphosphoproteins at each level of protein size. See legend to Fig. 4 for details. Analyses were performed for the phospho-PPI networks of “ALL” (A–C) and “Y2H” (E–F). Each bin corresponds to the protein length between *AA* and *AA*+100 (amino acids). See legend to Fig. 4 for details.
(0.44 MB TIF)

Figure S5 Comparison of node degree counts of phosphoproteins and nonphosphoproteins in terms of yeast functional annotations. The type of phospho-PPI network is “ALL.” For proteins corresponding to each Gene Ontology annotation of “biological process” (A), “molecular function” (B) and “cellular component” (C) in the *S. cerevisiae* GO Slim set, the average node degree of phosphoproteins divided by that of nonphosphoproteins is represented as the P to N (P/N) ratio.
(2.10 MB TIF)

Figure S6 Number counts of intersections between pair-wise protein relationships in the literature-derived signaling collection and in the PPI. For each category of PPI data, the number count of signaling reactions matched to protein interactions is represented by an arrow, along with *P* values estimated from negative controls generated by 10,000 repeats by RER. Gray bars represent the probability density distribution of the number count of the intersection observed using the negative controls generated by RER.
(0.24 MB TIF)

Figure S7 Cumulative probability distributions of node degrees of nonphosphoproteins, phosphoproteins with a single phosphosite, and phosphoproteins with two or more phosphosites in the phospho-PPI data of the “ALL” category. For each dataset, bars show proportions of proteins with more than the *k* interacting partners indicated on the horizontal axis [*P*≥(*k*)].
(0.90 MB TIF)

Figure S8 Number counts of interacting protein pairs of each phosphorylation pattern shown in the phospho-PPI networks of the “IMM” and “TAP” categories. See legend to Fig. 6 for details.
(0.60 MB TIF)

Figure S9 Number counts of IKMs formed in the “filtered” PPI network by each of the kinases shown in Fig. 7. For each kinase, arrows indicate number counts of IKMs formed by that kinase and the “filtered” PPI network, with *P* values estimated by comparison with negative controls. See legend to Fig. 7 for details.
(0.90 MB TIF)

Table S1 Phosphoproteome data used in this study.
(1.85 MB XLS)

Table S2 List of known and predicted *S. cerevisiae* genes and phosphorylation annotation.
(0.47 MB XLS)

Table S3 Phosphoproteome data used in this study.
(0.09 MB XLS)

## References

[1] Hunter T (2000). Signaling–2000 and beyond.. Cell.

[2] Manning G, Whyte DB, Martinez R, Hunter T, Sudarsanam S (2002). The protein kinase complement of the human genome.. Science.

[3] Pawson T, Nash P (2000). Protein-protein interactions define specificity in signal transduction.. Genes Dev.

[4] Aebersold R, Mann M (2003). Mass spectrometry-based proteomics.. Nature.

[5] Cravatt BF, Simon GM, Yates JR (2007). The biological impact of mass-spectrometry-based proteomics.. Nature.

[6] Huang PH, White FM (2008). Phosphoproteomics: unraveling the signaling web.. Mol Cell.

[7] Witze ES, Old WM, Resing KA, Ahn NG (2007). Mapping protein post-translational modifications with mass spectrometry.. Nat Methods.

[8] Beausoleil SA, Jedrychowski M, Schwartz D, Elias JE, Villen J (2004). Large-scale characterization of HeLa cell nuclear phosphoproteins.. Proc Natl Acad Sci U S A.

[9] Molina H, Horn DM, Tang N, Mathivanan S, Pandey A (2007). Global proteomic profiling of phosphopeptides using electron transfer dissociation tandem mass spectrometry.. Proc Natl Acad Sci U S A.

[10] Olsen JV, Blagoev B, Gnad F, Macek B, Kumar C (2006). Global, in vivo, and site-specific phosphorylation dynamics in signaling networks.. Cell.

[11] Sugiyama N, Masuda T, Shinoda K, Nakamura A, Tomita M (2007). Phosphopeptide enrichment by aliphatic hydroxy acid-modified metal oxide chromatography for nano-LC-MS/MS in proteomics applications.. Mol Cell Proteomics.

[12] Olsen JV, Vermeulen M, Santamaria A, Kumar C, Miller ML (2010). Quantitative phosphoproteomics reveals widespread full phosphorylation site occupancy during mitosis.. Sci Signal.

[13] Villen J, Beausoleil SA, Gerber SA, Gygi SP (2007). Large-scale phosphorylation analysis of mouse liver.. Proc Natl Acad Sci U S A.

[14] Chi A, Huttenhower C, Geer LY, Coon JJ, Syka JE (2007). Analysis of phosphorylation sites on proteins from *Saccharomyces cerevisiae* by electron transfer dissociation (ETD) mass spectrometry.. Proc Natl Acad Sci U S A.

[15] Ficarro SB, McCleland ML, Stukenberg PT, Burke DJ, Ross MM (2002). Phosphoproteome analysis by mass spectrometry and its application to *Saccharomyces cerevisiae*.. Nat Biotechnol.

[16] Holt LJ, Tuch BB, Villen J, Johnson AD, Gygi SP (2009). Global analysis of Cdk1 substrate phosphorylation sites provides insights into evolution.. Science.

[17] Gnad F, Ren S, Cox J, Olsen JV, Macek B (2007). PHOSIDA (phosphorylation site database): management, structural and evolutionary investigation, and prediction of phosphosites.. Genome Biol.

[18] Diella F, Gould CM, Chica C, Via A, Gibson TJ (2008). Phospho.ELM: a database of phosphorylation sites—update 2008.. Nucleic Acids Res.

[19] UniProt Consortium (2010). The Universal Protein Resource (UniProt) in 2010.. Nucleic Acids Res.

[20] Giot L, Bader JS, Brouwer C, Chaudhuri A, Kuang B (2003). A protein interaction map of *Drosophila melanogaster*.. Science.

[21] Han JD, Bertin N, Hao T, Goldberg DS, Berriz GF (2004). Evidence for dynamically organized modularity in the yeast protein-protein interaction network.. Nature.

[22] Ho Y, Gruhler A, Heilbut A, Bader GD, Moore L (2002). Systematic identification of protein complexes in *Saccharomyces cerevisiae* by mass spectrometry.. Nature.

[23] Ito T, Chiba T, Ozawa R, Yoshida M, Hattori M (2001). A comprehensive two-hybrid analysis to explore the yeast protein interactome.. Proc Natl Acad Sci U S A.

[24] Uetz P, Giot L, Cagney G, Mansfield TA, Judson RS (2000). A comprehensive analysis of protein-protein interactions in *Saccharomyces cerevisiae*.. Nature.

[25] Li S, Armstrong CM, Bertin N, Ge H, Milstein S (2004). A map of the interactome network of the metazoan *C. elegans*.. Science.

[26] Rual JF, Venkatesan K, Hao T, Hirozane-Kishikawa T, Dricot A (2005). Towards a proteome-scale map of the human protein-protein interaction network.. Nature.

[27] Tarassov K, Messier V, Landry CR, Radinovic S, Serna Molina MM (2008). An in vivo map of the yeast protein interactome.. Science.

[28] Barabasi AL, Albert R (1999). Emergence of scaling in random networks.. Science.

[29] Girvan M, Newman ME (2002). Community structure in social and biological networks.. Proc Natl Acad Sci U S A.

[30] Goh KI, Oh E, Jeong H, Kahng B, Kim D (2002). Classification of scale-free networks.. Proc Natl Acad Sci U S A.

[31] Watts DJ, Strogatz SH (1998). Collective dynamics of ‘small-world’ networks.. Nature.

[32] Hong EL, Balakrishnan R, Dong Q, Christie KR, Park J (2008). Gene Ontology annotations at SGD: new data sources and annotation methods.. Nucleic Acids Res.

[33] Salwinski L, Miller CS, Smith AJ, Pettit FK, Bowie JU (2004). The Database of Interacting Proteins: 2004 update.. Nucleic Acids Res.

[34] Ivanic J, Yu X, Wallqvist A, Reifman J (2009). Influence of protein abundance on high-throughput protein-protein interaction detection.. PLoS One.

[35] von Mering C, Krause R, Snel B, Cornell M, Oliver SG (2002). Comparative assessment of large-scale data sets of protein-protein interactions.. Nature.

[36] Yu H, Zhu X, Greenbaum D, Karro J, Gerstein M (2004). TopNet: a tool for comparing biological sub-networks, correlating protein properties with topological statistics.. Nucleic Acids Res.

[37] Newman JR, Ghaemmaghami S, Ihmels J, Breslow DK, Noble M (2006). Single-cell proteomic analysis of *S. cerevisiae* reveals the architecture of biological noise.. Nature.

[38] Dosztanyi Z, Chen J, Dunker AK, Simon I, Tompa P (2006). Disorder and sequence repeats in hub proteins and their implications for network evolution.. J Proteome Res.

[39] Dunker AK, Cortese MS, Romero P, Iakoucheva LM, Uversky VN (2005). Flexible nets. The roles of intrinsic disorder in protein interaction networks.. FEBS J.

[40] Haynes C, Oldfield CJ, Ji F, Klitgord N, Cusick ME (2006). Intrinsic disorder is a common feature of hub proteins from four eukaryotic interactomes.. PLoS Comput Biol.

[41] Gsponer J, Futschik ME, Teichmann SA, Babu MM (2008). Tight regulation of unstructured proteins: from transcript synthesis to protein degradation.. Science.

[42] Landry CR, Levy ED, Michnick SW (2009). Weak functional constraints on phosphoproteomes.. Trends Genet.

[43] Tan CS, Bodenmiller B, Pasculescu A, Jovanovic M, Hengartner MO (2009). Comparative analysis reveals conserved protein phosphorylation networks implicated in multiple diseases.. Sci Signal.

[44] Taylor IW, Linding R, Warde-Farley D, Liu Y, Pesquita C (2009). Dynamic modularity in protein interaction networks predicts breast cancer outcome.. Nat Biotechnol.

[45] Tan CS, Jørgensen C, Linding R (2010). Roles of “junk phosphorylation” in modulating biomolecular association of phosphorylated proteins?. Cell Cycle.

[46] Walhout AJ, Vidal M (2001). Protein interaction maps for model organisms.. Nat Rev Mol Cell Biol.

[47] Fiedler D, Braberg H, Mehta M, Chechik G, Cagney G (2009). Functional organization of the *S. cerevisiae* phosphorylation network.. Cell.

[48] Linding R, Jensen LJ, Ostheimer GJ, van Vugt MA, Jørgensen C (2007). Systematic discovery of in vivo phosphorylation networks.. Cell.

[49] Gong W, Zhou D, Ren Y, Wang Y, Zuo Z (2008). PepCyber:P∼PEP: a database of human protein protein interactions mediated by phosphoprotein-binding domains.. Nucleic Acids Res.

[50] Yachie N, Saito R, Sugahara J, Tomita M, Ishihama Y (2009). In silico analysis of phosphoproteome data suggests a rich-get-richer process of phosphosite accumulation over evolution.. Mol Cell Proteomics.

[51] Maslov S, Sneppen K (2002). Specificity and stability in topology of protein networks.. Science.

[52] Walhout AJ, Reboul J, Shtanko O, Bertin N, Vaglio P (2002). Integrating interactome, phenome, and transcriptome mapping data for the *C. elegans* germline.. Curr Biol.

[53] Ptacek J, Devgan G, Michaud G, Zhu H, Zhu X (2005). Global analysis of protein phosphorylation in yeast.. Nature.

[54] Imamura H, Yachie N, Saito R, Ishihama Y, Tomita M (2010). Towards the systematic discovery of signal transduction networks using phosphorylation dynamics data.. BMC Bioinformatics.

[55] Murray DB, Beckmann M, Kitano H (2007). Regulation of yeast oscillatory dynamics.. Proc Natl Acad Sci U S A.

[56] Masuda T, Tomita M, Ishihama Y (2008). Phase transfer surfactant-aided trypsin digestion for membrane proteome analysis.. J Proteome Res.

[57] Rappsilber J, Ishihama Y, Mann M (2003). Stop and go extraction tips for matrix-assisted laser desorption/ionization, nanoelectrospray, and LC/MS sample pretreatment in proteomics.. Anal Chem.

[58] Rappsilber J, Mann M, Ishihama Y (2007). Protocol for micro-purification, enrichment, pre-fractionation and storage of peptides for proteomics using StageTips.. Nat Protoc.

[59] Kyono Y, Sugiyama N, Imami K, Tomita M, Ishihama Y (2008). Successive and selective release of phosphorylated peptides captured by hydroxy acid-modified metal oxide chromatography.. J Proteome Res.

[60] Ishihama Y, Rappsilber J, Andersen JS, Mann M (2002). Microcolumns with self-assembled particle frits for proteomics.. J Chromatogr A.

[61] Shimizu K, Muraoka Y, Hirose S, Tomii K, Noguchi T (2007). Predicting mostly disordered proteins by using structure-unknown protein data.. BMC Bioinformatics.

