# Supplementary figures and images for: Integrative Features of the Yeast Phosphoproteome and Protein–Protein Interaction Map
Source: PLoS Comput Biol. 2011 Jan 27;7(1):e1001064. doi: 10.1371/journal.pcbi.1001064 (PMC3029238; doi:10.1371/journal.pcbi.1001064)

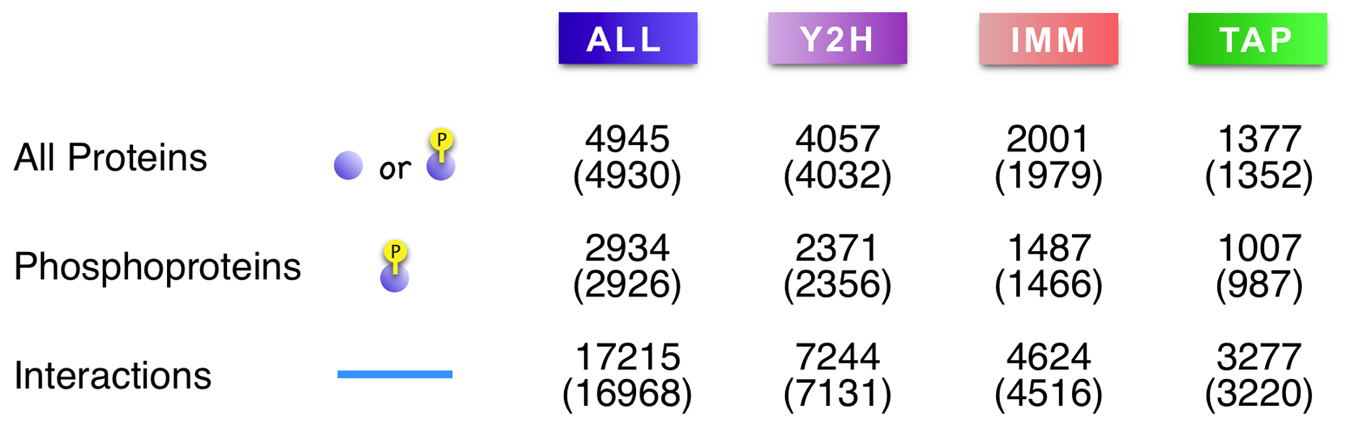

Supplement: Figure S1 — Contents of the phospho-PPI network generated for each experimental method used in the PPI assay. Numbers in parentheses indicate those derived by the “filtered” network. (1.73 MB TIF) [file pcbi.1001064.s001.tif]

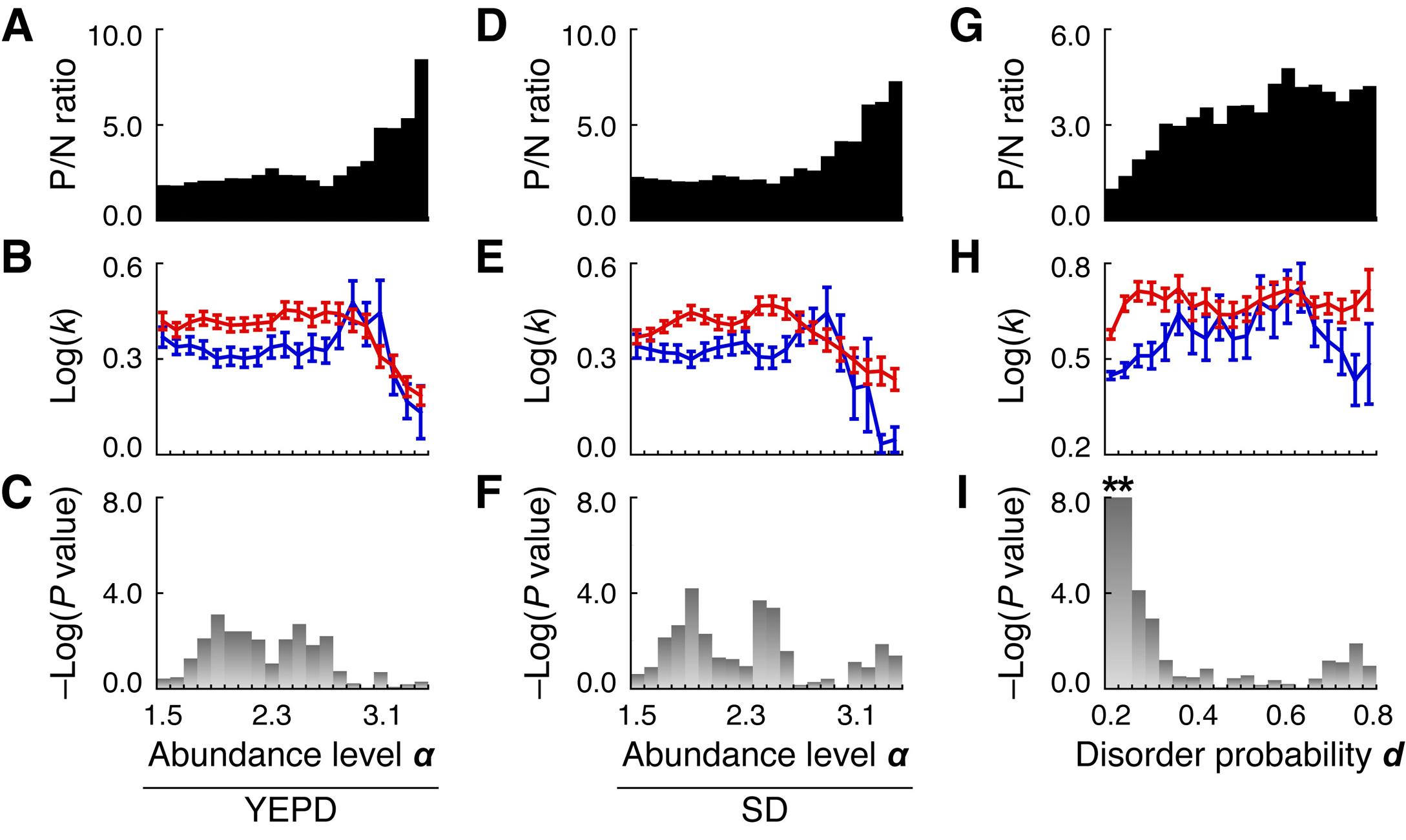

Supplement: Figure S2 — Differences between node degree levels of phosphoproteins and nonphosphoproteins of the “Y2H” phospho-PPI network at each level of protein abundance or protein disorder. See legend to Fig. 4 for details. (0.67 MB TIF) [file pcbi.1001064.s002.tif]

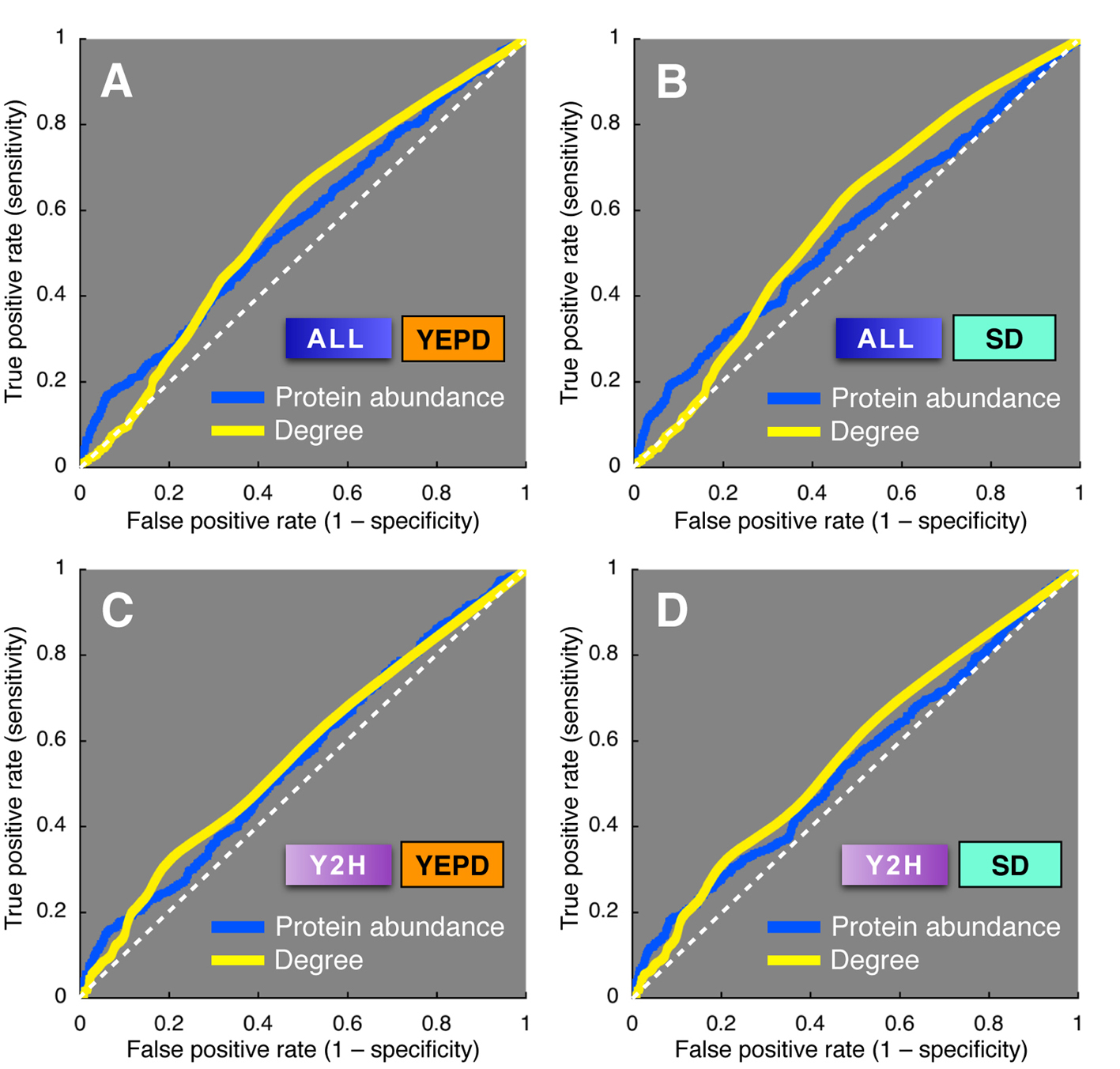

Supplement: Figure S3 — Comparison of abilities to predict phosphoproteins by node degree level and protein abundance level. (A) “ALL” PPI data and proteome abundance data measured for cells grown in YEPD medium. (B) “ALL” PPI and proteome abundance for cells grown in SD medium. (C) “Y2H” PPI and proteome abundance for cells grown in YEPD medium. (D) “Y2H” PPI and proteome abundance for cells grown in SD medium. For each predictor, the true-positive rate or “sensitivity” (defined here as the fraction of phosphoproteins correctly predicted to be phosphoproteins) and the false-positive rate or “1 – specificity” (defined here as the fraction of nonphosphoproteins incorrectly predicted to be phosphoproteins) are shown at a series of score thresholds. (5.75 MB TIF) [file pcbi.1001064.s003.tif]

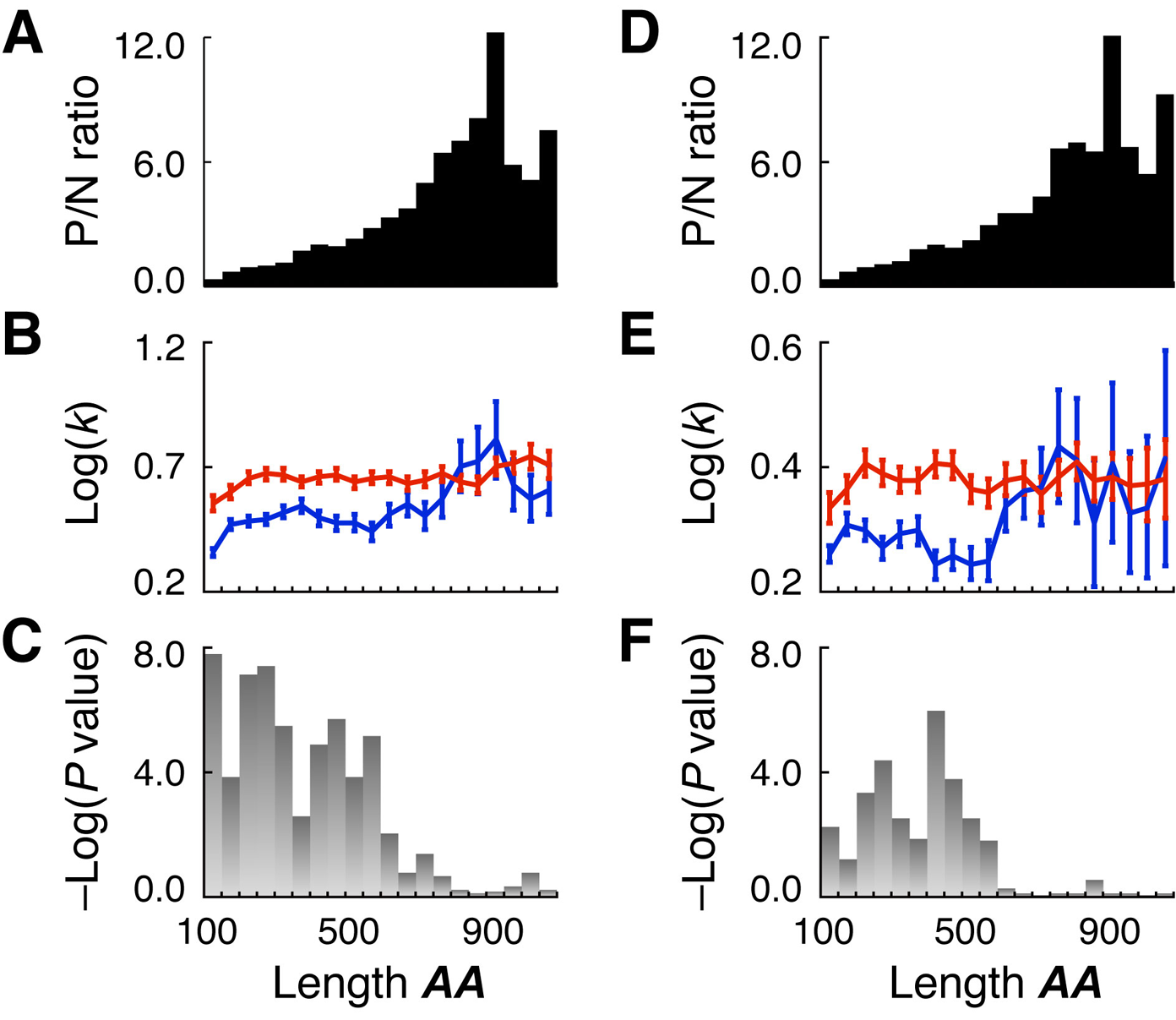

Supplement: Figure S4 — Differences between node degree levels of phosphoproteins and nonphosphoproteins at each level of protein size. See legend to Fig. 4 for details. Analyses were performed for the phospho-PPI networks of “ALL” (A–C) and “Y2H” (E–F). Each bin corresponds to the protein length between AA and AA+100 (amino acids). See legend to Fig. 4 for details. (0.44 MB TIF) [file pcbi.1001064.s004.tif]

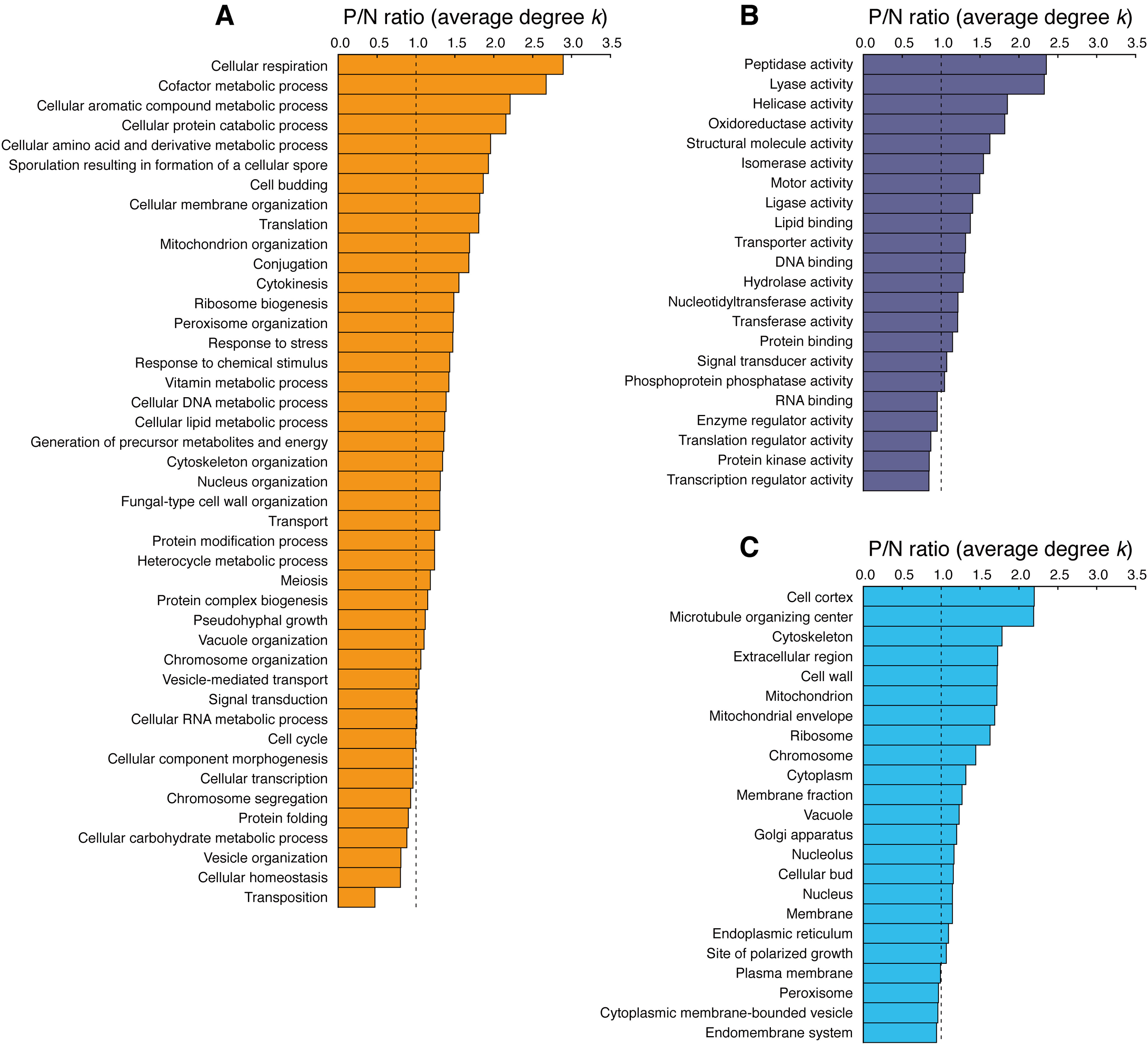

Supplement: Figure S5 — Comparison of node degree counts of phosphoproteins and nonphosphoproteins in terms of yeast functional annotations. The type of phospho-PPI network is “ALL.” For proteins corresponding to each Gene Ontology annotation of “biological process” (A), “molecular function” (B) and “cellular component” (C) in the S. cerevisiae GO Slim set, the average node degree of phosphoproteins divided by that of nonphosphoproteins is represented as the P to N (P/N) ratio. (2.10 MB TIF) [file pcbi.1001064.s005.tif]

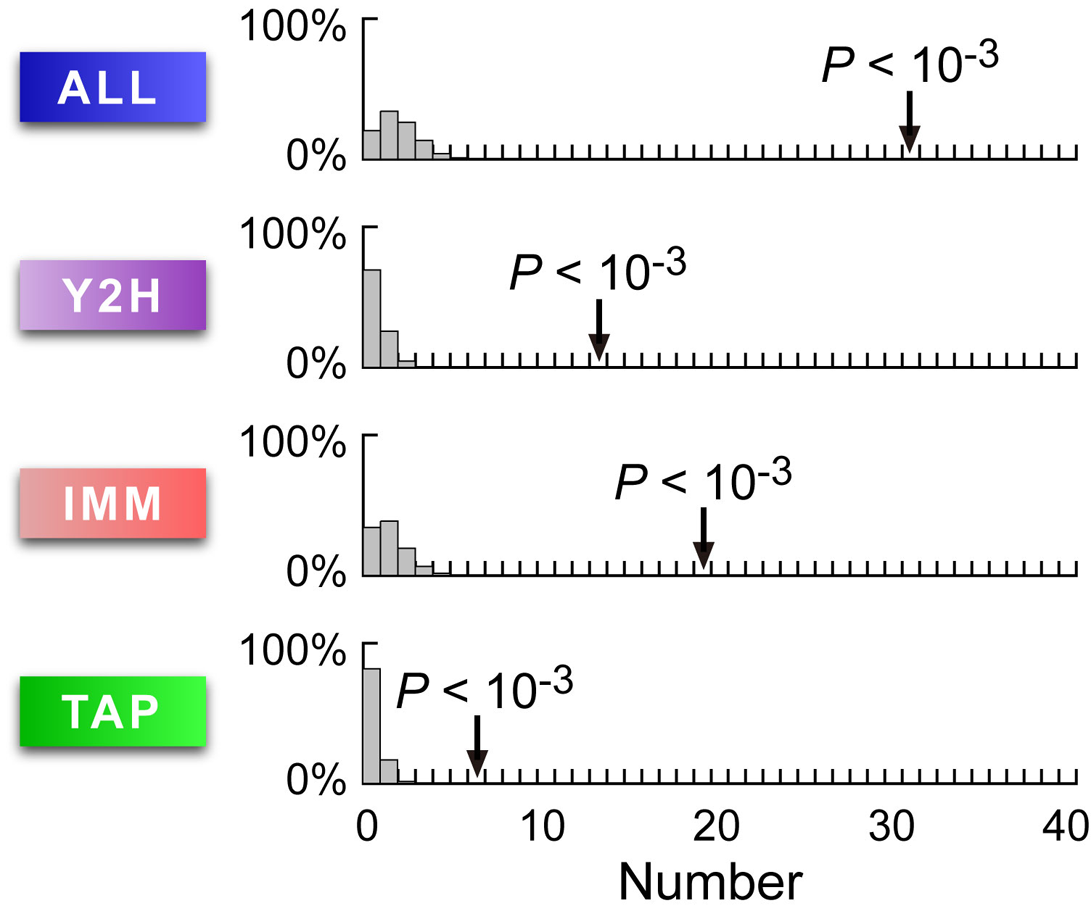

Supplement: Figure S6 — Number counts of intersections between pair-wise protein relationships in the literature-derived signaling collection and in the PPI. For each category of PPI data, the number count of signaling reactions matched to protein interactions is represented by an arrow, along with P values estimated from negative controls generated by 10,000 repeats by RER. Gray bars represent the probability density distribution of the number count of the intersection observed using the negative controls generated by RER. (0.24 MB TIF) [file pcbi.1001064.s006.tif]

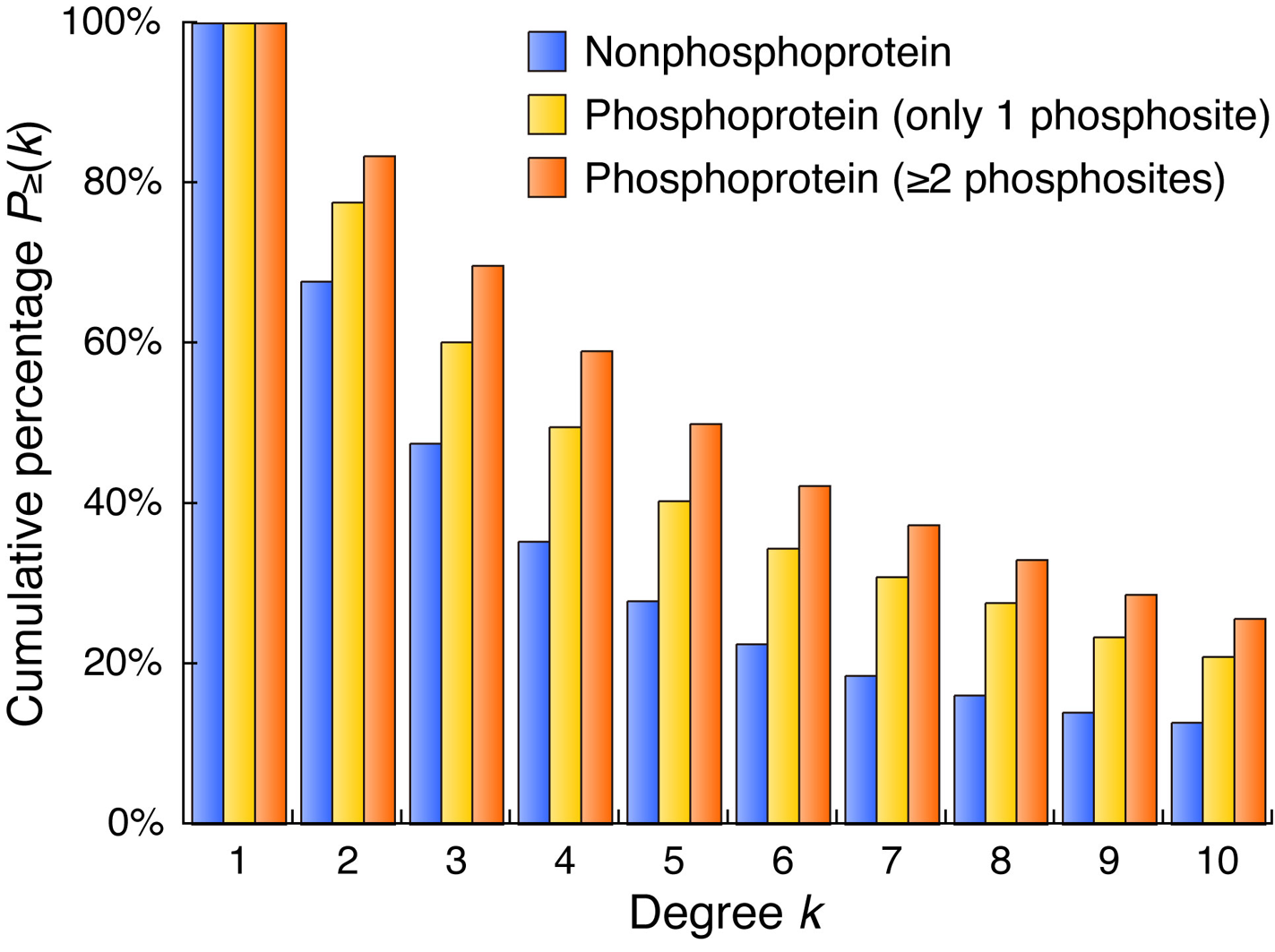

Supplement: Figure S7 — Cumulative probability distributions of node degrees of nonphosphoproteins, phosphoproteins with a single phosphosite, and phosphoproteins with two or more phosphosites in the phospho-PPI data of the “ALL” category. For each dataset, bars show proportions of proteins with more than the k interacting partners indicated on the horizontal axis [P≥(k)]. (0.90 MB TIF) [file pcbi.1001064.s007.tif]

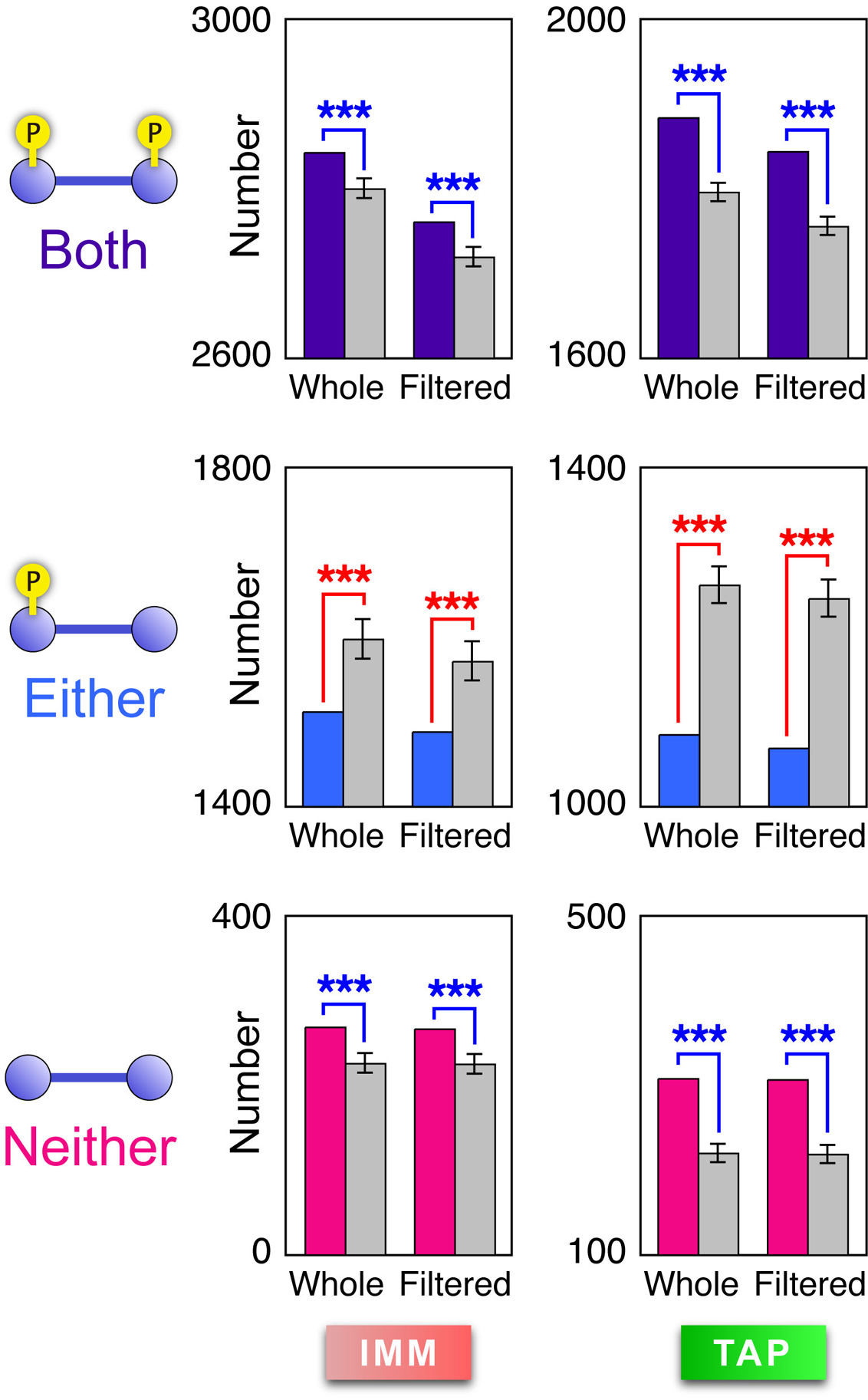

Supplement: Figure S8 — Number counts of interacting protein pairs of each phosphorylation pattern shown in the phospho-PPI networks of the “IMM” and “TAP” categories. See legend to Fig. 6 for details. (0.60 MB TIF) [file pcbi.1001064.s008.tif]

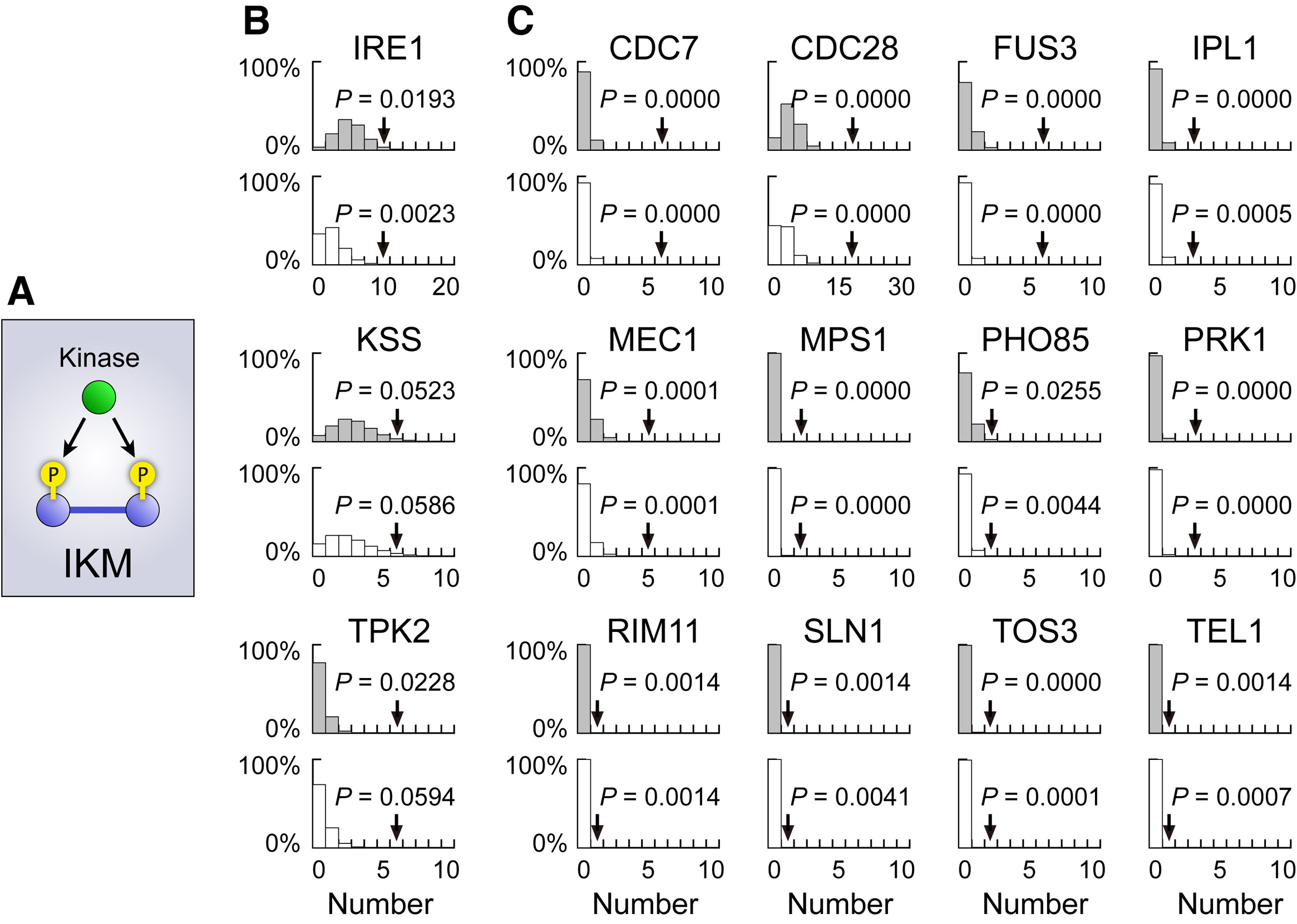

Supplement: Figure S9 — Number counts of IKMs formed in the “filtered” PPI network by each of the kinases shown in Fig. 7. For each kinase, arrows indicate number counts of IKMs formed by that kinase and the “filtered” PPI network, with P values estimated by comparison with negative controls. See legend to Fig. 7 for details. (0.90 MB TIF) [file pcbi.1001064.s009.tif]
